# Management impact of ^18^F-DCFPyL PET/CT in hormone-sensitive prostate cancer patients with biochemical recurrence after definitive treatment: a multicenter retrospective study

**DOI:** 10.1007/s00259-021-05222-5

**Published:** 2021-02-05

**Authors:** Dennie Meijer, Pim J. van Leeuwen, Pepijn M. J. Oosterholt, Yves J. L. Bodar, Henk G. van der Poel, N. Harry Hendrikse, Maarten L. Donswijk, Maurits Wondergem, Annelies E. Vellekoop, R. Jeroen A. van Moorselaar, Jakko A. Nieuwenhuijzen, Daniela E. Oprea-Lager, André N. Vis

**Affiliations:** 1 Department of Urology, Prostate Cancer Network the Netherlands, Amsterdam University Medical Center, VU University, De Boelelaan 1117, 1081 Amsterdam, HV The Netherlands; 2 Department of Radiology and Nuclear Medicine, Cancer Center Amsterdam, Amsterdam University Medical Center, VU University, Amsterdam, The Netherlands; 3 Department of Urology, Prostate Cancer Network the Netherlands, The Netherlands Cancer Institute, Amsterdam, The Netherlands; 4 Department of Clinical Pharmacology and Pharmacy, Amsterdam University Medical Center, VU University, Amsterdam, The Netherlands; 5 Department of Nuclear Medicine, Noordwest Ziekenhuisgroep, Alkmaar, The Netherlands; 6 Department of Urology, Amstelland Hospital, Amstelveen, The Netherlands

**Keywords:** Biochemical recurrence, Change of management, ^18^F-DCFPyL PET/CT, Prostate cancer, PSMA

## Abstract

**Purpose:**

The aim of this study was to investigate whether an early, accurate identification of disease using ^18^F-DCFPyL PET/CT imaging resulted in a change of decision on treatment management, for individual patients with biochemically recurrent (BCR), hormone-sensitive prostate cancer.

**Methods:**

In this retrospective study, a total of 253 patients with BCR who underwent restaging ^18^F-DCFPyL PET/CT were assessed. Two urologists specialized in uro-oncology were asked to formulate a preferred treatment for each patient before and after knowing the results of the ^18^F-DCFPyL PET/CT.

**Results:**

Out of 253 patients, 191 (75%) underwent robot-assisted radical prostatectomy (RARP) as primary therapy, and 62 (25%) external beam radiation therapy (EBRT). In 103/253 cases (40.7%), a preferred treatment change based on the ^18^F-DCFPyL PET/CT findings was reported. In patients post-RARP, a positive ^18^F-DCFPyL PET/CT (OR 6.21; 95%CI 2.78–13.8; *p* < 0.001) and positive pathological lymph node status (pN1) (OR 2.96; 95%CI 1.15–7.60; *p* = 0.024) were significant predictors for an intended change of management, whereas a positive surgical margin (OR 0.42; 95%CI 0.20–0.88; *p* = 0.022) was inversely associated with an intended change of management.

**Conclusion:**

In this study, we found a significant impact of ^18^F-DCFPyL PET/CT on the intended management of patients with biochemically recurrent hormone-sensitive prostate cancer. A positive ^18^F-DCFPyL PET/CT scan, positive pathological lymph node status, and a negative surgical margin status were significantly associated with increased odds of having a change of management based on ^18^F-DCFPyL PET/CT findings.

**Supplementary Information:**

The online version contains supplementary material available at 10.1007/s00259-021-05222-5.

## Introduction

Prostate cancer (PCa) is associated with increasing age. In elderly men it is the second most frequent malignancy after lung cancer [1]. Robot-assisted laparoscopic radical prostatectomy (RARP) and external beam radiation therapy (EBRT) are two important curative treatment options for localized PCa. However, 20–50% of patients who have undergone RARP or EBRT will experience biochemical recurrence (BCR) of disease within 10 years [2–6].

Since the introduction of prostate-specific membrane antigen (PSMA) positron emission tomography/computed tomography (PET/CT), the detection of metastases in patients with BCR at low prostate-specific antigen (PSA) values has improved substantially [7, 8]. PSMA is a transmembrane protein which is overexpressed in malignant prostatic tissue [9, 10]. In addition to the widely used ^68^Gallium-PSMA-11 [11], ^18^F-DCFPyL is a promising novel, second-generation ^18^F-radiolabeled tracer [12]. Both radiotracers demonstrated improved detection of metastases compared to conventional imaging techniques, such as computed tomography (CT), bone scan, or magnetic resonance imaging (MRI) [8, 13, 14]. Moreover, multiple studies showed a high diagnostic accuracy of PSMA PET/CT imaging in patients with BCR, with a variable positive predictive value of 78–99% [15, 16].

When a modern imaging technique is associated with an enhanced diagnostic accuracy, it is expected that the treatment strategy will change due to an improved staging [17]. Indeed, Calais et al. [18] showed that a change in management was seen in 54 out of 101 (53%) patients who underwent ^68^Ga-PSMA PET/CT for BCR after curative therapy for PCa. Comparably, Song et al. [19] showed a similar percentage of patients (60%) with a change of disease management for ^18^F-DCFPyL PET/CT in a small cohort of 72 patients with BCR after RARP or EBRT. However, as both studies used actual implemented management as outcome instead of the intended treatment based on PSMA PET/CT, other factors such as patient preferences might have influenced the results. Consequently, if these management decisions were truly based on the PSMA PET/CT findings remains unclear. Therefore, the present study investigated the role of modern imaging for PCa in a large cohort of patients, in whom disease management was assessed both with and without the knowledge of the outcome of ^18^F-DCFPyL PET/CT, in patients with BCR after RARP or EBRT.

## Materials and methods

This retrospective study was conducted by the Prostate Cancer Network the Netherlands, i.e., Amsterdam UMC VU University (VUmc), the Netherlands Cancer Institute (NCI), and the Noordwest Ziekenhuisgroep (NWZ) Alkmaar, in the period December 2016 to December 2019. Approval of the institutional review board of VUmc (VUmc2020.048) and NCI (IRBd19-182) was obtained for this study, waiving the need to receive informed consent. All patients included from NWZ have given written informed consent.

### Inclusion and exclusion criteria of patients

In this study, we included 253 consecutive patients with biochemically recurrent, hormone-sensitive PCa, after RARP or EBRT, who underwent an ^18^F-DCFPyL PET/CT between December 2016 and December 2019. BCR was defined as a PSA level ≥ 0.2 ng/mL after RARP [20] and > 2.0 ng/mL after EBRT [21]. Patients with rising PSA values after RARP or EBRT, and who did not meet the criteria for BCR but still underwent ^18^F-DCFPyL PET/CT for restaging purposes, were also included in this analysis.

**Fig. 1:**
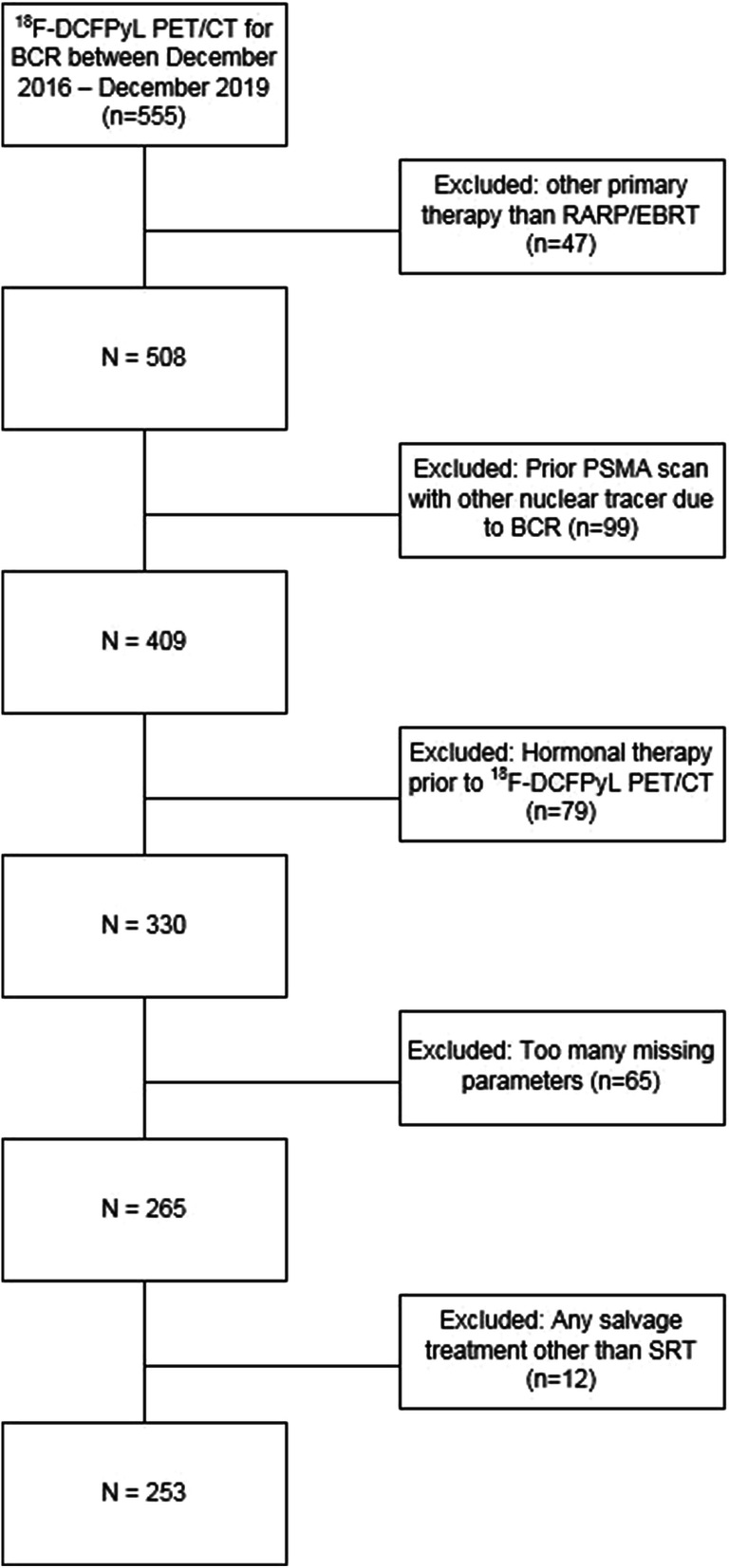
Flowchart of all screened patients on eligibility

Patients who underwent primary therapy, other than RARP or EBRT (e.g., brachytherapy, focal therapy of the prostate), were excluded. Also patients who priorly underwent PSMA-based imaging for BCR with a tracer other than ^18^F-DCFPyL such as ^68^Ga-PSMA-11 or ^18^F-PSMA-1007 were excluded. Moreover, patients who had received adjuvant androgen deprivation therapy (ADT), any hormonal therapy (HT) at the time of performing ^18^F-DCFPyL PET/CT, or any salvage treatment other than salvage radiation therapy (SRT) after RARP were not eligible for this analysis. Lastly, patients with incomplete clinical data were excluded from the study (Fig. 1).

### Assessment on intended treatment of individual cases before and after ^18^F-DCFPyL

**Fig. 2:**
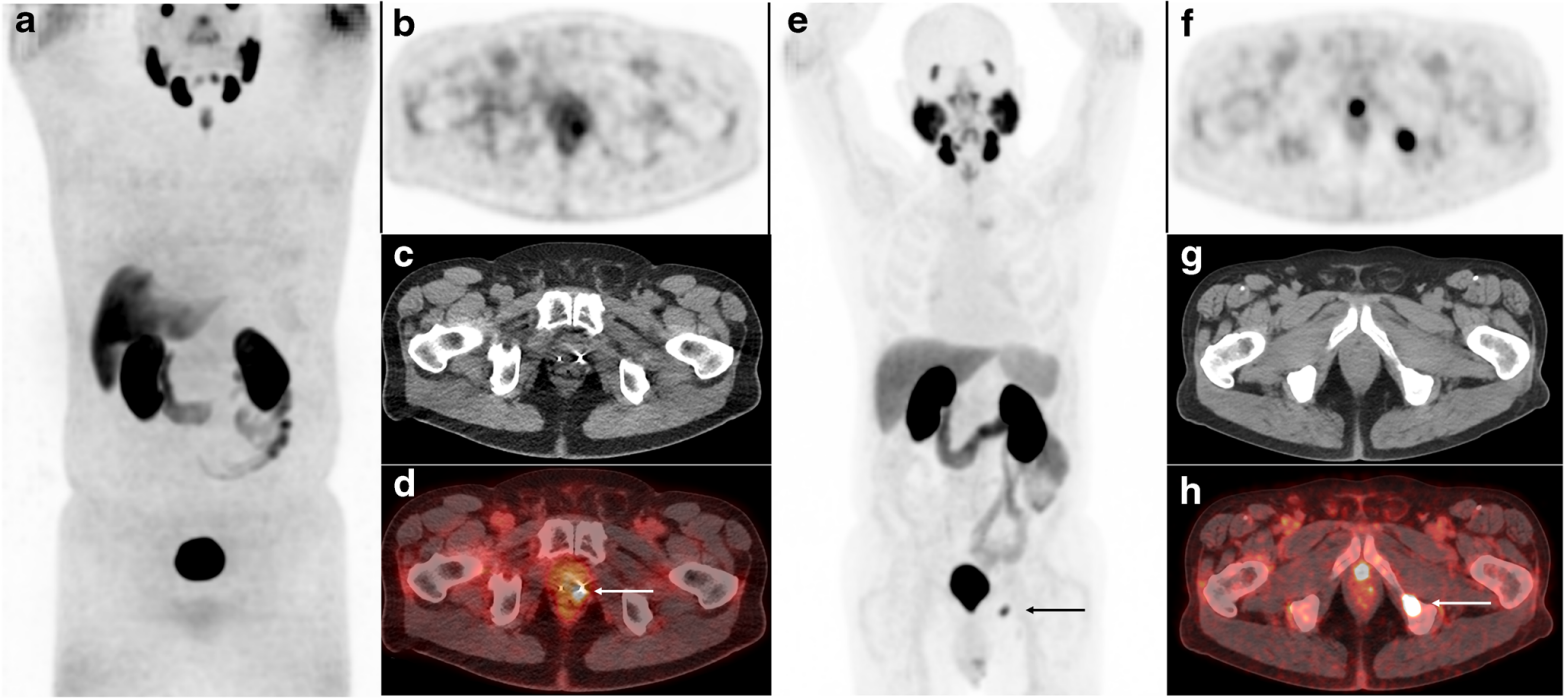
PSMA PET/CT images (maximum intensity projection (MIP), left panel; PET, upper panel; CT, mid-panel; fused PET/CT, lower panel) in 2 patients with BCR. **a–d** Patient 1: A 67-year-old patient, with a rising PSA of 1.1 ng/mL, 5 years after EBRT (PSA-nadir 0.1 ng/mL). (Delayed) step-up (systemic) hormonal therapy was chosen as the preferred treatment. However, the performed PSMA PET/CT showed local recurrent disease (miTr), resulting in a change of management from systemic treatment to local treatment. **e–h** Patient 2: A 76-year-old patient, with a rising PSA of 0.8 ng/mL, 2 years after RARP (PSA nadir <0.1 ng/mL). Salvage radiation therapy to the prostatic fossa (local treatment) was chosen as the preferred treatment. However, the performed PSMA PET/CT showed one bone metastasis (miM1b) in the left os ischium, resulting in a change of management from local treatment to metastasis-directed therapy

Two urologists (AV, PvL), specialized in PCa surgery and care at the Amsterdam UMC and at the NCI, assessed all individual cases with BCR after RARP or EBRT. Anonymized patient charts, including patients’ clinical, pathological, and biochemical characteristics, were presented to both urologists independently by two medical researchers (DM, PO). An intended treatment advice was given for all cases, firstly without the knowledge of the results of the ^18^F-DCFPyL PET/CT and, secondly, with the findings of ^18^F-DCFPyL imaging. Thereafter, a consensus meeting was organized in which all cases and patient histories were rediscussed, and a definitive treatment advice was given for every individual case Fig. 2.

After collecting the selected treatment advices, four different treatment categories were described, based on their clinical value (i.e., 1 to 4), further subdivided into a total of nine treatment allocations (i.e., a to i; Table 1): (1) local treatment (salvage radiotherapy (RT) to the prostatic fossa, with or without HT (a), salvage focal therapy (b)); (2) locoregional treatment (whole pelvis RT with (c) or without (d) salvage RT to the prostatic fossa, with or without HT, and salvage pelvic lymph node dissection (sPLND) (e); (3) metastasis-directed radiotherapy (MDT) of distant (oligo)metastatic lesions (f); and (4) systemic treatment ((delayed) step-up HT (g), chemotherapy (h), and a combination of both(i)).

**Table 1:** Treatment options in patients with BCR, subclassified into treatment categories

Treatment options	Category
Radiotherapy prostatic fossa ± hormonal therapy	Local
Salvage focal therapy	Local
Radiotherapy pelvic area ± hormonal therapy	Locoregional
Radiotherapy prostatic fossa + pelvic area ± hormonal therapy	Locoregional
Salvage lymph node dissection	Locoregional
Metastasis-directed therapy	MDT
(Delayed) step-up hormonal therapy	Systemic
Hormonal therapy + chemotherapy	Systemic
Chemotherapy	Systemic

### Patient data and patient charts

All demographic data (age at the time of treatment, age at the time of performing the PSMA PET/CT), clinical parameters (clinical tumor stage, history of PCa treatment), biochemical parameters (initial PSA level, PSA level at time of scan), radiological data (^18^F-DCFPyL PET/CT findings at the time of BCR), and pathological data (biopsy tumor features, pathological T-stage, radical prostatectomy Grade Group (GG), surgical margin status, pathological lymph node status) were collected for all patients. Furthermore, for patients who underwent EBRT, the number of fractions, dose, and number of months of hormonal therapy were reported. For each case, previous urological or radiation oncology treatments (such as salvage RT) were recorded, as was the biochemical follow-up of patients who underwent RARP or EBRT.

### ^18^F-DCFPyL PET/CT imaging

At Amsterdam UMC, location VUmc, imaging was performed using a Philips Ingenuity TF (Philips Healthcare®, the Netherlands/USA) PET/CT system. ^18^F-DCFPyL was synthesized via direct radiofluoration at the on-site cyclotron facility, compliant to good manufacturing practices (GMP [22, 23]). The median tracer dose administered was 311 MBq (interquartile range (IQR) 301–322 MBq). PET images were acquired approximately 120 min after intravenous injection. The scan trajectory included mid-thigh to skull base, with 4 min per bed position.

At NCI, imaging was performed using a Philips Gemini TF-II or Vereos Digital PET/CT (Philips Healthcare®, the Netherlands/USA). ^18^F-DCFPyL was administered as an intravenous bolus injection with a median dose of 197 MBq (IQR 189–207 MBq). Scanning commenced after an incubation period of approximately 60 min, with 2 min per bed position over the complete scan range.

At NWZ, imaging was performed using a Siemens Biograph TruePoint-16 (Siemens Healthineers, Germany) PET/CT scanner. Scanning was performed after approximately 120 min, with a median tracer dose of 290 MBq (IQR 280–323 MBq). PET images were made mid-thigh to skull base, with 5 min per bed position.

PET images were combined with either a low-dose CT scan (120–140 kV, 30–80 mAs with dose modulation) or a diagnostic CT scan (130 kV, 110mAs). All PET images were corrected for scatter, decay, and random coincidences; attenuation correction was performed using CT images.

### Image interpretation of ^18^F-DCFPyL PET/CT imaging

Interpretation of the scans was performed by nuclear medicine physicians in the three hospitals, all of whom have an ample experience with PSMA PET reading (>400 scans). PSMA reporting was performed in accordance with the PROMISE criteria [24]. If at least one metastatic lesion or sign of local recurrence in the prostate/prostatic fossa (miTr) was found, the scan was considered to be positive (i.e., focal and higher uptake of the PSMA tracer compared to the surrounding tissue, not compatible with physiological uptake). Loco-regional lymph node metastases in the true pelvis were classified as miN1. miM1a was defined as lymph node metastatic disease outside the surgical template, whereas lesions that showed increased PSMA expression in the bones or the visceral organs were classified as miM1b and miM1c, respectively. This classification was conform the EAU guidelines [20].

### Statistical analysis

Descriptive statistics, median, and interquartile range (IQR) were used to summarize numerical variables, whereas percentages (%) were used for categorical variables. The differences in change of management rates for PSMA-positive and PSMA-negative patients, as well as the difference between different therapies (RARP, RARP + SRT, and EBRT), were compared using the Chi-square test. Statistical significance was set at *p* < 0.05 [25].

In order to investigate the potential association between selected variables and the likelihood that would lead to a change of management of ^18^F-DCFPyL PET/CT, a multivariable logistic regression was performed. Multiple parameters that could potentially predict change of management were studied: PSA value at time of the scan, ^18^F-DCFPyL PET/CT findings, RARP GG, surgical margin status, pathological lymph node status, pathological T-stage, and the administration of salvage therapies prior to scan. The dichotomous outcome variable was change of management based on ^18^F-DCFPyL PET/CT imaging. As multiple pathological variables were included, the multivariable analysis was solely performed on the RARP group.

## Results

### Patient characteristics

Out of 253 included patients, 191 (75%) underwent RARP as primary therapy, and 25% (62 patients) EBRT. In patients who underwent RARP, simultaneous extended pelvic lymph node dissection (ePLND) was performed in 118 out of 191 cases (62%). In men who were treated by EBRT, additional hormonal therapy was given in 52 out of 62 cases (84%). Hormonal treatment was ended in all cases at a median of 31 months (IQR 12–49) prior to PSMA PET/CT. SRT prior to restaging ^18^F-DCFPyL PET/CT for BCR was given in 41 out of 191 patients (21%) who underwent RARP (Table 2).

**Table 2:** Baseline characteristics of all included patients with BCR

Patient characteristics	post-RARP(*n* = 150)	post-RARP + SRT(*n* = 41)	post-EBRT (*n* = 62)	*p* value
Age at the time of ^18^F-DCFPyL PET/CT, years; median (IQR)	69 (64–73)	67 (64–72)	72 (67–77)	**0.002**
Initial PSA at diagnosis, ng/mL; median (IQR)	9.4 (6.9–16.1)	11.0 (7.8–20.3)	18.1 (9.4–45.1)	**<0.001**
Time between primary therapy and ^18^F-DCFPyL PET/CT,months; median (IQR)	22 (9–51)	74 (46–95)	58 (39–80)	**<0.001**
Clinical T-stage, *n* (%)				
cT1–cT2	131 (87)	37 (90)	29 (47)	**<0.001**
cT3–cT4	13 (9)	4 (10)	32 (52)	
Unknown	6 (4)	0 (0)	1 (1)	
Biopsy Grade Group according to ISUP, *n* (%)				
1–2 (Gleason score 3 + 3 = 6 and 3 + 4 = 7)	76 (51)	28 (68)	21 (34)	**0.01**
3 (Gleason score 4 + 3 = 7)	30 (20)	5 (12)	13 (21)	
4–5 (Gleason score ≥ 8)	44 (29)	8 (20)	28 (45)	
Additional hormonal treatment, *n* (%)				
No	–	–	10 (16)	–
Yes			52 (84)	
RARP Grade Group according to ISUP, n (%)				
1–2 (Gleason score 3 + 3 = 6 and 3 + 4 = 7)	49 (33)	12 (29)	–	0.72
3 (Gleason score 4 + 3 = 7)	52 (34)	17 (42)		
4–5 (Gleason score ≥ 8)	49 (33)	12 (29)		
Surgical margin status, *n* (%)				
Negative	65 (44)	11 (27)	–	0.06
Positive	80 (53)	30 (73)		
Unknown	5 (3)	0 (0)		
Pathological lymph node status, *n* (%)				
pN0	49 (33)	18 (44)	–	**0.02**
pN1	47 (31)	4 (10)		
pNx	54 (36)	19 (46)		
Pathological T-stage, *n* (%)				
pT2	57 (38)	17 (42)	–	0.82
pT3a	48 (32)	11 (27)		
≥pT3b	45 (30)	13 (31)		

Significant *p*-values are shown in bold

*RARP* robot-assisted laparoscopic radical prostatectomy, *EBRT* external beam radiation therapy, *PSA* prostate-specific antigen, *ISUP* International Society of Urological Pathology

### ^18^F-DCFPyL PET/CT findings

The time interval between RARP and ^18^F-DCFPyL PET/CT was median 22 months (IQR 9–51), compared to 74 months (IQR 46–95) between RARP + SRT and ^18^F-DCFPyL PET/CT and median 58 months (IQR 39–80) between EBRT and ^18^F-DCFPyL-based imaging (*p* < 0.001; Table 2). Out of 253 ^18^F-DCFPyL PET/CT scans, 167 (66%) scans were reported positive, and 86 (34%) showed no evidence of disease (NED). The median PSA level at the time of the PET scan post-RARP was 0.5 ng/mL (IQR 0.2–1.1), median 0.9 ng/mL (IQR 0.3–2.8) in patients post-RARP + SRT, and median 2.8 ng/mL (IQR 1.3–5.6) in patients post-EBRT. Local recurrence of disease in the prostatic fossa (miTr) only was found 34 times (13%), PSMA-positive lymph nodes in the pelvic area (miN1) were found in 23% (57/253) of cases, isolated extra-pelvic PSMA-positive lymph nodes (miM1a) accounted for 2 cases (1%), whereas bone or visceral metastases (miM1b–miM1c) were found in 18 patients (6%). In 58/253 cases (23%), multiple locations showed increased ^18^F-DCFPyL uptake (Table 3).

**Table 3:** ^18^F-DCFPyL PET/CT findings, stratified per location

	post-RARP (*n* = 150)	post-RARP + SRT (*n* = 41)	post-EBRT (*n* = 62)
^18^F-DCFPyL PET/CT findings, *n* (%)			
Negative for cancer	65 (44)	12 (29)	9 (15)
Local recurrence of disease (miTr)	17 (11)	3 (8)	14 (23)
Locoregional lymph node metastases (miN1)	32 (21)	12 (29)	13 (21)
Distant lymph node metastases (miM1a)	2 (1)	0 (0)	0 (0)
Bone or visceral metastases (miM1b–miM1c)	10 (7)	2 (5)	4 (6)
Multiple locations	24 (16)	12 (29)	22 (35)
^18^F-DCFPyL PET/CT findings, stratified per location, *n* (%)			
Negative	65 (44)	12 (29)	9 (15)
Inside the pelvis (miTr/miN1)	56 (37)	19 (46)	27 (43)
Outside the pelvis (≥miM1)	12 (8)	2 (5)	7 (11)
Inside and outside the pelvis	17 (11)	8 (20)	19 (31)
^18^F-DCFPyL PET/CT findings, extent of metastatic disease, *n* (%)			
Negative/local recurrence (miTr)	82 (55)	15 (36)	23 (37)
Unimetastastic disease	27 (18)	10 (24)	3 (5)
Oligometastatic disease (2–5 metastases)	25 (17)	8 (20)	21 (34)
Polymetastatic disease (>5 metastases)	16 (11)	8 (20)	15 (24)

*RARP* robot-assisted laparoscopic radical prostatectomy, *SRT* salvage radiation therapy, *EBRT* external beam radiation therapy, *PET* positron emission tomography, *CT* computed tomography

### Impact on patient management

The treatment advice for all cases, both in absence and presence of the findings of ^18^F-DCFPyL imaging, is presented in Table 4. Treatment categories and treatment allocations were given for the different clinical BCR indications. In 103 out of 253 cases (40.7%), restaging ^18^F-DCFPyL PET/CT scan findings were reason to change treatment advice. For patients who were advised to undergo local treatment based on an unknown result of ^18^F-DCFPyL PET/CT, treatment changed to locoregional, MDT, or systemic treatment in 8/86, (9%), 13/86 (15%), and 9/86 patients (10%), respectively, based on the ^18^F-DCFPyL PET/CT findings. In patients where systemic treatment was proposed based on the clinical findings (in the absence of ^18^F-DCFPyL PET/CT imaging), 13/158 (8%) cases, 39/158 (25%) cases, and 21/158 (13%) cases had an intended treatment change based on ^18^F-DCFPyL PET/CT findings to local treatment, locoregional treatment, and MDT, respectively. A total of 85/158 (54%) cases remained on systemic treatment even after PSMA-based imaging.

**Table 4:** Selection of cases in which ^18^F-DCFPyL PET/CT findings resulted in an intended change of management

Clinical situation of BCR	Number of patients	Without ^18^F-DCFPyL treatment advice	^18^F-DCFPyL findings	After ^18^F-DCFPyL treatment advice	Change of management
RARP + pNx + PSA ≤1.0 ng/mL	38	Local (37)	NED (17)	Local (22)	15/38 = 39%
		Systemic (1)	miTr (5)	Locoregional (5)	
			miN1 (8)	MDT (7)	
			miM1a–miM1c (4)	Systemic (4)	
			Multiple locations (4)		
RARP + pN1 + PSA ≤1.0 ng/mL		Locoregional (8)	NED (15)	Locoregional (30)	24/35 = 69%
		Systemic (27)	miTr (4)	MDT (2)	
	35		miN1 (10)	Systemic (3)	
			miM1a–miM1c (1)		
			Multiple locations (5)		
RARP + pN0 + PSA ≤1.0 ng/mL		Local (37)	NED (26)	Local (28)	9/38 = 24%
		Systemic (1)	miTr (3)	Locoregional (2)	
	38		miN1 (5)	MDT (4)	
			miM1a–miM1c (3)	Systemic (4)	
			Multiple locations (1)		
RARP + SRT		Systemic (41)	NED (12)	Locoregional (4)	13/41 = 32%
			miTr (3)	MDT (9)	
	41		miN1 (12)	Systemic (28)	
			miM1a–miM1c (2)		
			Multiple locations (12)		
EBRT + PSA ≥2.0 ng/mL		Systemic (42)	NED (4)	Local (7)	17/42 = 40%
			miTr (10)	Locoregional (6)	
	42		miN1 (8)	MDT (4)	
			miM1a–miM1c (5)	Systemic (25)	
			Multiple locations (15)		

*BCR* biochemical recurrence, *RARP* robot-assisted laparoscopic radical prostatectomy, *PSA* prostate-specific antigen, *NED* no evidence of disease, *MDT* metastasis-directed radiation therapy, *SRT* salvage radiation therapy, *EBRT* external beam radiation therapy

The proportion of patients in whom management of disease was changed was not statistically different between men undergoing RARP (44.0%), men undergoing RARP + SRT (31.7%), and those patients who underwent EBRT (38.7%), as initial treatment (χ2; *p* = 0.34).

### Predictors of management changes

In order to analyze a homogenous group, only RARP patients have been taken into account. On multivariable logistic regression analysis, a positive ^18^F-DCFPyL PET/CT scan (odds ratio (OR) 6.21; 95%CI 2.78–13.8; *p* < 0.001) or a positive pathological lymph node status (OR 2.96; 95%CI 1.15–7.60; *p* = 0.024) was significant predictors for an intended change of management, whereas a positive surgical margin (OR 0.42; 95%CI 0.20–0.88; *p* = 0.022) was inversely associated with an intended change of management. The PSA level at the time of the scan, pathological T-stage, RARP GG, and the administration of SRT were not associated with an intended management change (**Supplementary Table** **1**).

## Discussion

In the present study, we systematically assessed the impact of ^18^F-DCFPyL PET/CT imaging on the change of management of patients with biochemically recurrent hormone-sensitive PCa after RARP or EBRT. The medical charts of 253 patients were presented to two separate urologists specialized in uro-oncology, who chose from various treatment options, initially without knowledge of PSMA-based imaging and subsequently with the results of restaging ^18^F-DCFPyL PET/CT. Patients who underwent RARP as primary curative therapy had a treatment management change based on ^18^F-DCFPyL PET/CT findings in 44.0% (66/150 patients) at a median PSA level at the time of the scan of 0.5 ng/mL (IQR 0.2–1.1), compared to 31.7% (13/41 patients) in patients who underwent both RARP + SRT with a median PSA at performing the ^18^F-DCFPyL PET/CT of 0.9 ng/mL (IQR 0.3–2.8) and 38.7% (24/62 patients) in patients who priorly underwent EBRT with a median PSA at the time of the scan of 2.8 ng/mL (IQR 1.3–5.6).

Previous studies that assessed the impact of ^68^Ga-PSMA-11 PET/CT on PCa management found promising results for the proportion of changes in disease management. Albisinni et al. [26] found a change of management in 76% (99/131) of patients who underwent ^68^Ga-PSMA-11 PET/CT for BCR after radical prostatectomy or multimodality treatment. One of the main differences to our study is that a total of 14 different treatment options for relapse were used compared to only 4 treatment categories in our study. Due to this higher number of treatment options, a change of management was more likely to occur. Moreover, continuing surveillance (withholding hormonal therapy) was often chosen as treatment option in that study. Therefore, patients in whom systemic treatment was proposed pre-PSMA, and active surveillance post-PSMA, were considered as having a change of management.

In our study, surveillance was pooled with (delayed) step-up hormonal therapy to assess actual change of management instead of merely delaying treatment options until further notice. Lastly, while the percentage of patients who underwent RARP as primary curative therapy was almost comparable to that of our series (i.e., 81% versus 75% in our study), the median PSA level at the time of PSMA-based imaging in their study was substantially higher (2.2 ng/mL) compared to the median PSA in the present cohort (0.8 ng/mL). Therefore, it is likely that the proportion of positive PSMA scans was substantially higher in their study [13].

Another study that assessed the impact of ^68^Ga-PSMA-11 PET/CT on treatment plan is that of Calais et al. [18]. A change of management in 53% of included patients (54/101) was reported, especially in patients with positive ^68^Ga-PSMA-11 PET/CT scans. Similar to the study of Albisinni et al. [26], Calais et al. reported on 9 different treatment options, including active surveillance. In addition, patients with BCR were scanned at a median PSA level of 1.7 ng/mL (range, 0.05–140 ng/mL), again a substantially higher level than that in our study (0.8 ng/mL). Moreover, the management options consisted of the preferred treatment before the PSMA scan and the actually implemented treatment for the patient. Since Calais et al. applied the actual implemented management as outcome instead of the intended treatment, which was used in our study, other factors such as patient preferences might have influenced the results.

Moreover, two recent studies evaluated the impact of ^68^Ga-PSMA-11 PET/CT on radiotherapeutic management, both at initial staging and at BCR. Koerber et al. [27] found a change of radiotherapeutic management in 50.8% of patients, whereas Sterzing et al. [28] observed a change of management in 56.3% of patients with BCR after RARP. In both studies, the PSA level at the time of the scan was higher (1.1 ng/mL and 2.8 ng/mL, respectively) compared to that in the present study (0.8 ng/mL), which may explain the slightly higher percentages of management changes in those studies. Next to these studies, Sonni et al. [29] confirmed the impact of ^68^Ga-PSMA PET/CT on staging and management of PCa patients outside of the two main classical indications (BCR and presurgical staging).

Only one previous study assessed the impact of the ^18^F-DCFPyL tracer on the change of management in patients with BCR after prostatectomy or radiotherapy. Song et al. [19] found a change in management in 60% of cases (43/72 patients) on BCR. Interestingly, in 59 patients, conventional imaging results, such as those from bone scan, CT, or MRI, were also available, and these were compared to ^18^F-DCFPyL findings. From the cases who underwent any of the other staging imaging modalities as well as PSMA PET/CT, 17 out of 43 had lesion localization on ^18^F-DCFPyL PET only, despite negative results on conventional imaging. The most important methodological difference between that study and the present study was that Song et al. reported on the actual implemented management post ^18^F-DCFPyL PET/CT, which may have influenced their rate of change of management. Furthermore, the median PSA at the time of ^18^F-DCFPyL PET/CT was 3.0 ng/mL, which was substantially higher than in the present study, probably resulting in a higher percentage positive PSMA PET/CT scans, which may lead to a higher percentage of change of management.

To assess whether clinical, biochemical, or pathological parameters could predict intended management changes in patients with BCR, a multivariable analysis was performed. A positive ^18^F-DCFPyL PET/CT scan (miTr, miN1, miM1a–miM1a c), positive pathological lymph node status (pN1), and a negative surgical margin status (R0) were significantly associated with an increased odds of intended treatment management change based on the ^18^F-DCFPyL PET/CT scan. Patients with a negative pathological lymph node status (pN0) and a positive surgical margin status (R1) were less likely to have a management change based on ^18^F-DCFPyL PET/CT findings. Possibly, patients in whom ^18^F-DCFPyL PET/CT findings did not result in an intended management change may be withheld an ^18^F-DCFPyL PET/CT. Future studies on which patients to withhold PSMA imaging are warranted to assess this issue.

It needs to be addressed that a treatment change due to the application of modern imaging modalities does not necessarily translate into improved oncological outcomes. Through the earlier detection of recurrences and metastases, the Will Rogers phenomenon is likely to occur, i.e., the improvement of clinical outcome in separate staging groups, whereas the prognosis in the entire group is not changed [17]. The concept of the Will Rogers phenomenon and its pitfalls was reported for different other malignancies and might well be observed in the pro-PSMA trial [14]. In this prospective, randomized clinical trial assessing the diagnostic accuracy of PSMA PET/CT imaging in the diagnostic setting, including 302 patients with high-risk PCa, Hofman et al. assigned patients at random to conventional imaging with CT and bone scanning or ^68^Ga-PSMA-11 PET/CT. PSMA-based imaging proved to be more accurate than conventional imaging regarding the detection of pelvic nodal and distant metastatic disease, and treatment changes were more frequently observed with modern PET imaging. Despite this improved diagnostic accuracy, disease-free survival or overall survival was not (yet) assessed in the trial. Although PSMA PET is increasingly implemented as the “standard of care,” the effect of increased diagnostic accuracy on oncological outcome should be better clarified.

Several limitations need to be addressed. Firstly, all included patients underwent an ^18^F-DCFPyL PET/CT in three different hospitals, with different PET scanners, different scan protocols, and different nuclear medicine physicians reporting. This might have resulted in heterogeneous results. Secondly, the inherent personal interpretation of the European and Dutch urology guidelines by both urologists may result in different preferred treatment options inter-individually and from country to country. MDT, for instance, accounting for 13% of the intended management changes, is still considered experimental in the EAU guidelines for patients with oligometastatic disease [20]. Moreover, all patients included in this study underwent ^18^F-DCFPyL PET/CT as a restaging modality. Consequently, no conventional imaging techniques were used. It could be that a selection of lesions visualized by PSMA PET/CT would have been found by conventional imaging techniques. Lastly, due to the retrospective nature of this analysis, a bias may have occurred. To truly determine the impact of PSMA PET/CT imaging on management decisions, future randomized trials are warranted.

## Conclusion

This study showed a significant impact of ^18^F-DCFPyL PET/CT on intended management of patients with biochemically recurrent hormone-sensitive PCa. A positive ^18^F-DCFPyL PET/CT scan, a positive pathological lymph node status, and a negative surgical margin status were significantly associated with increased odds of having a change of management based on ^18^F-DCFPyL PET/CT findings. Whether treatment changes by modern PSMA-based imaging result in an improvement in oncological outcomes is a question that needs to be answered within well-designed prospective trials. In conclusion, this study demonstrates that ^18^F-DCFPyL PET/CT might be a helpful tool to obtain the best management decisions in patients with biochemically recurrent PCa.

## Supplementary information

ESM 1 (DOCX 14 kb)
