# Supplementary material for: Management impact of 18F-DCFPyL PET/CT in hormone-sensitive prostate cancer patients with biochemical recurrence after definitive treatment: a multicenter retrospective study
Source: Eur J Nucl Med Mol Imaging. 2021 Feb 5;48(9):2960–9. doi: 10.1007/s00259-021-05222-5 (PMC8263452; doi:10.1007/s00259-021-05222-5)
Supplement: Supplementary file 1 — (DOCX 14 kb) [file 259_2021_5222_MOESM1_ESM.docx]

**Article title:** Management Impact of ^18^F-DCFPyL PET/CT in Prostate Cancer Patients with Biochemical Recurrence after curative treatment

**Journal name:** Eur J Nucl Med Mol Imag

Dennie Meijer^a,b^, Pim J. van Leeuwen^c^, Pepijn M. J. Oosterholt^a^,

Yves J.L. Bodar^a,b^, Henk G. van der Poel^c^, N. Harry Hendrikse^b,d^,

Maarten L. Donswijk^c^, Maurits Wondergem^e^, Annelies E. Vellekoop^f^,

R. Jeroen A. van Moorselaar^a^, Jakko A. Nieuwenhuijzen^a,c^,

Daniela E. Oprea-Lager^b^, André N. Vis^a,c^

^a^ Amsterdam University Medical Center, VU University, Department of Urology, Prostate Cancer Network the Netherlands, Amsterdam, The Netherlands
^b^ Amsterdam University Medical Center, VU University, Department of Radiology & Nuclear Medicine, Cancer Center Amsterdam, Amsterdam, The Netherlands
^c^ The Netherlands Cancer Institute, Department of Urology, Prostate Cancer Network the Netherlands, Amsterdam, The Netherlands
^d^ Amsterdam University Medical Center, VU University, Department of Clinical Pharmacology and Pharmacy, Amsterdam, The Netherlands
^e^ Noordwest Ziekenhuisgroep, Alkmaar, Department of Nuclear Medicine, Alkmaar, The Netherlands
^f^ Amstelland Hospital, Department of Urology, Amstelveen, The Netherlands

Corresponding author

Dennie Meijer, MD
ORCID 0000-0002-8298-575X
De Boelelaan 1117, 1081 HV Amsterdam
Telephone: +31-20-4443289 / FAX: +31-20-4446031
d.meijer2@amsterdamumc.nl

**Supplementary Table 1.** Multivariable logistic regression analysis on the impact on the change of management of ^18^F-DCFPyL PET/CT in patients with BCR

|  | **Odds ratio (95% CI)** | ***p*-value** |
| --- | --- | --- |
| **PSA at the time of the scan (continuous)** | 0.89 (0.78-1.01) | 0.07 |
|  |  |  |
| **^18^F-DCFPyL PET/CT findings** |  |  |
| Negative | Reference |  |
| Positive | 6.21 (2.78-13.8) | **<0.001** |
|  |  |  |
| **Pathological T-stage** |  |  |
| pT2 | Reference |  |
| pT3a | 1.30 (0.53-3.21) | 0.56 |
| ≥pT3b | 1.93 (0.75-4.99) | 0.18 |
|  |  |  |
| **RARP Grade Group according to ISUP** |  |  |
| 1 - 2 (Gleason Score 3+3=6 and 3+4=7) | Reference |  |
| 3 (Gleason Score 4+3=7) | 0.69 (0.29-1.64) | 0.40 |
| 4 - 5 (Gleason Score ≥8) | 0.74 (0.28-1.93) | 0.53 |
|  |  |  |
| **Pathological lymph node status** |  |  |
| pN0 | Reference |  |
| pN1 | 2.96 (1.15-7.60) | **0.024** |
| pNx | 2.38 (0.99-5.67) | 0.051 |
|  |  |  |
| **Surgical margin status** |  |  |
| Negative | Reference |  |
| Positive | 0.42 (0.20-0.88) | **0.022** |
|  |  |  |
| **Salvage radiation therapy prior to scan** |  |  |
| No | Reference |  |
| Yes | 0.69 (0.29-1.68) | 0.42 |
